# Macrophage polarization toward M1 phenotype in T cell transfer colitis model

**DOI:** 10.1186/s12876-023-03054-1

**Published:** 2023-11-27

**Authors:** Shin Ebihara, Toshiki Urashima, Wataru Amano, Hideto Yamamura, Noriko Konishi

**Affiliations:** Biological/Pharmacological Research Laboratories, Takatsuki Research Center, Central Pharmaceutical Research Institute, Japan Tobacco, Inc, 1-1 Murasaki-cho, Takatsuki, Osaka 569-1125 Japan

**Keywords:** T cell transfer colitis model, Macrophage, M1/M2 markers

## Abstract

**Background:**

T cell transfer colitis model is often used to study the CD4^+^ T cell functions in the intestine. However, the specific roles of macrophages in colitis remain unclear. In this study, we aimed to evaluate the phenotype and functions of macrophages in the colonic lamina propria (LP) in a colitis model.

**Methods:**

Colitis was induced in scid mice via the adaptive transfer of CD4^+^CD45RB^hi^ T cells. Then, flow cytometry was used to determine the number of macrophages in the colonic LP and expression of cytokines in macrophages at the onset of colitis. Moreover, M1/M2 macrophage markers were detected in the colonic LP during colitis development using high-dimensional single-cell data and gating-based analyses. Expression levels of M1 markers in macrophages isolated from the colonic LP were measured using quantitative reverse transcription-polymerase chain reaction. Additionally, macrophages were co-cultured with T cells isolated from the colon to assess colitogenic T cell activation.

**Results:**

Infiltration of macrophages into the colon increased with the development of colitis in the T cell transfer colitis model. M1/M2 macrophage markers were observed in this model, as observed in the colon of patients with inflammatory bowel disease (IBD). Moreover, number of M1 macrophages increased, whereas that of M2 macrophages decreased in the colonic LP during colitis development. M1 macrophages were identified as the main source of inflammatory cytokine production, and colitogenic T cells were activated via interactions with these macrophages.

**Conclusions:**

Our findings revealed that macrophages polarized toward the M1 phenotype in LP during colitis development in the T cell transfer colitis model. Therefore, the colitis model is suitable for the evaluation of the efficacy of macrophage-targeted drugs in human IBD treatment. Furthermore, this model can be used to elucidate the in vivo functions of macrophages in the colon of patients with IBD.

**Supplementary Information:**

The online version contains supplementary material available at 10.1186/s12876-023-03054-1.

## Background

Inflammatory bowel disease (IBD), including Crohn’s disease (CD) and ulcerative colitis (UC), is a group of chronic relapsing disorders of the gastrointestinal tract that are pathologically characterized by intestinal inflammation and epithelial injury [1, 2]. Recently, a “treat-to-target” approach has been proposed for mucosal healing [3]. However, the efficacy of many drugs used in clinical therapies remains inadequate [4, 5]. Therefore, a deeper insight into the pathogenic mechanisms is needed to identify the dysregulated immune pathways that lead to tissue inflammation in patients with IBD. Murine models of IBD are useful in investigating the pathogenic mechanisms of human IBD.

Several mouse models of experimental colitis, which are suggested to mimic human IBD, have been used to investigate the immunopathogenesis of IBD [6–8]. In particular, the T cell transfer colitis model exhibits high resemblance to the chronic nature of human IBD than the erosive and self-limiting models of acute colitis in terms of histopathological features [7] and gene expression patterns [9]. Therefore, this colitis model is often used to evaluate the therapeutic effects of several drugs and drug candidates in IBD treatment [7, 10]. As the development of colitis in this model is induced by the transfer of naïve CD4^+^ T cells to lymphopenic recipients, it is often used for the study of CD4^+^ T cell functions in the intestine [11–13]. However, many other immune cells, such as inflammatory monocytes, macrophages, and neutrophils, are also involved in colitis pathogenesis in this model. Interleukin (IL)-10 signaling by innate immune cells plays a critical role in the regulation of mucosal homeostasis, independent of effector T cells, in the T cell transfer colitis model [14]. However, the specific roles of innate immune cells in the T cell transfer colitis model remain unknown. The number of macrophages (innate immune cells), especially pro-inflammatory M1 macrophages, is markedly increased in the inflamed mucosa of patients with IBD [15–17]. Upon tissues damage, the innate immune system detects damage-associated molecular patterns (DAMPs) and pathogen-associated molecular patterns (PAMPs) and attracts circulating neutrophils. Infiltrating neutrophils then recruit inflammatory monocytes to mount an appropriate response to the inflammogen. During intestinal inflammation, the terminal differentiation of inflammatory monocytes to mature intestinal macrophages is disrupted. The balance shifts more towards an M1 macrophages, which maintain their pro-inflammatory capacity by the secretion of inflammatory cytokines including TNFα and p40, promoting Th1 and/or Th17 immune responses and aggravating tissue damage [15–17]. Since accumulating evidence strongly supports the idea that enforcing a pro-resolving (anti-inflammatory) phenotype of macrophages (M2 macrophages) might be a novel therapeutic approach to control intestinal inflammation and restore function, many studies have investigated the functions of M1 macrophages in the bowel of patients with IBD [15–17].

In this study, we investigated the phenotype and functions of macrophages in the colonic Lamina propria (LP) of a T cell transfer colitis model for extrapolation to human IBD. Our results suggest that the number of mucosal M1 (pro-inflammatory) macrophages increase, whereas that of mucosal M2 (anti-inflammatory) macrophages, which are present in a steady state, decrease during colitis development.

## Materials and methods

### Mice

Female C.B-17/Icr-Prkdc < scid>/CrlCrlj (scid) mice were purchased from the Jackson Laboratory (Kanagawa, Japan), and female BALB/c mice were purchased from CLEA Japan (Tokyo, Japan). All mice were maintained under specific pathogen-free conditions at a room temperature of 23 ± 3 °C and air humidity of 55 ± 15% on a 12-h/12-h light/dark cycle. All experiments were conducted in accordance with ARRIVE guidelines and in compliance with the Guidelines for Animal Experimentation of the Central Pharmaceutical Research Institute of Japan Tobacco, Inc. (Protocol No. 02800, Data: Jan 18, 2022).

### T cell transfer colitis model

Colitis was induced as previously described [18]. Briefly, CD4^+^ T cell suspensions were prepared from the spleens of BALB/c mice using the MACS CD4^+^ T Cell Isolation Kit (Miltenyi Biotech, Bergisch Gladbach, Germany). Naive T cells were isolated with a Cell Sorter SH800S (Sony Biotechnology, Tokyo, Japan) using two antibodies against CD4 (Thermo Fisher Scientific, Waltham, MA, USA) and CD45RB (Thermo Fisher Scientific). Mice were intraperitoneally inoculated with 2 × 10^5^ CD4^+^CD45RB^hi^ T cells. The sham-treated mice were injected with phosphate-buffered saline (Thermo Fisher Scientific).

### Colonoscopy and histology

For colonoscopy, mice were anesthetized with isoflurane (Viatris, Tokyo, Japan), and feces were removed by injecting saline through a flexible feeding tube (Fuchigami, Kyoto, Japan). A rigid telescope (diameter, 1.9 mm; Smith and Nephew, London, UK) was rectally inserted into the mice for 4 cm using the Olympus CLH-250 Xenon Light Source (Olympus, Tokyo, Japan) as previously described [19]. During endoscope withdrawal, a video of the colon was recorded using the Olympus Video System OTV-SC2 (Olympus) and TEAC UR-4MD Medical Video Recorder (TEAC, Tokyo, Japan). Then, colonoscopic findings were scored as follows: mucosal thickening (0 = transparent, 1 = moderate, 2 = marked, and 3 = non-transparent), vasculature (0 = normal, 1 = moderate, 2 = marked, and 3 = absent/bleeding), and granularity (0 = none, 1 = moderate, 2 = marked, and 3 = extreme) [20]. For microscopic examination, colons were removed within 24 h of endoscopy after killing the mice under anesthesia. The colons were cut longitudinally, divided into three sections (proximal, middle, and distal), and fixed in 10% neutral-buffered formalin (FUJIFILM Wako Pure Chemical, Osaka, Japan). The tissues were embedded in paraffin wax and stained with hematoxylin and eosin. Tissue specimens for pathological evaluation were prepared by the Biopathology Institute (Kokuto, Japan). The three sections of colons were scored according to the histological criteria as follows: epithelial hyperplasia (0 = none, 1 = < 1.25-fold increase in epithelial length, 2 = 1.25–1.5-fold increase in epithelial length, 3 = 1.6–2-fold increase in epithelial length, and 4 = > 2-fold increase in epithelial length), goblet cell and crypt loss (0 = none, 1 = < 25%, 2 = 25–50%, 3 = 51–75%, and 4 = 76–100%), and inflammatory infiltration (0 = none, 1 = ≤ 25% increased presence in mucosa, 2 = > 25% increased presence in mucosa, 3 = < 25% infiltration in submucosa, 4 = < 75% infiltration in submucosa, and 5 = > 75% submucosa infiltration and sometimes transmural infiltration) [21]. Colonoscopy and histology findings were scored by two investigators who were blinded to the treatment.

### Measurement of colonic cytokine levels

Colonic segments were rinsed with saline, blotted dry, and stored at − 80 °C. The segments were homogenized using beads in distilled water containing Sample Diluent Concentrate 1 (R&D Systems, Minneapolis, MN, USA) and a protease inhibitor cocktail (Sigma-Aldrich, St Louis, MO, USA). After centrifugation at 12,000 g for 10 min at 4 °C to remove the debris, protein concentration was determined using a DC protein assay kit (Bio-Rad Laboratories, Richmond, CA, USA). Colonic cytokine levels were quantified using the enzyme-linked immunosorbent assay (ELISA) kits for tumor necrosis factor (TNF)-α (R&D Systems), p40 (Aviva Systems Biology, San Diego, CA, USA), monocyte chemoattractant protein (MCP)-1 (R&D Systems), and macrophage inflammatory protein (MIP)-1α (R&D Systems), according to the manufacturers’ instructions.

### Preparation and culture of colonic cells

LP cells were isolated from the colon, as previously described [12]. Briefly, colons were cut longitudinally, washed in cold Hank’s balanced salt solution containing 2% fetal bovine serum (FBS), penicillin (100 units/mL), and streptomycin (100 units/mL), and minced. The tissues were completely digested at 37 °C for 40 min with gentle stirring in the RPMI 1640 medium containing 2% FBS, penicillin (100 units/mL), and streptomycin (100 units/mL) and supplemented with collagenase D (100 U/mL; Sigma-Aldrich), and DNase (20 mg/mL; Roche Diagnostics, Basel, Switzerland). LP cells were purified on a 40/75% Percoll gradient via centrifugation at 600 g for 20 min at 25 °C. Cells were then incubated for 4 h in the presence of brefeldin A (Thermo Fisher Scientific).

### Flow cytometry analysis

Antibodies against CD45 were purchased from BD Biosciences (San Jose, CA, USA). Antibodies against CD11b, Ly6G, Ly6C, TCRβ, CD11c, CD206, CD335 (NKp46), F4/80, TNFα, p40, and IL-10 were purchased from BioLegend (San Diego, CA, USA). Cell surface staining was performed according to standard techniques after treatment with the anti-CD16/32 antibody (BD Biosciences) to block FcγR binding. Dead cells were excluded using the Fixable Viability Dye eFluor 780 (FVD780; Thermo Fisher Scientific). For intracellular staining, the cells were stained with different cell surface antibodies, fixed, permeabilized, and intracellularly stained for TNFα, p40, or IL-10. Gating strategies were set with reference to the isotype or fluorescence minus one (FMO) control. Flow cytometry was performed using the BD LSRFortessa X-20 flow cytometer (BD Biosciences), and data were analyzed using the FlowJo software, version 10.8.0 (BD Biosciences). Cytobank computational tool visualization of t-stochastic Neighbor Embedding (viSNE) was used for cluster analysis. To generate viSNE maps, samples were uploaded to Cytobank, and 80,000 live CD45^+^ cells were subsampled from the data. After sub-sampling, viSNE was performed using various parameters (F4/80, CD11c, and CD206). Finally, viSNE maps were visualized using the Cytobank interface, which was used to generate figures (color coding by marker expression levels).

### RNA isolation and reverse transcription quantitative polymerase chain reaction (RT-qPCR)

Total RNA was prepared from macrophages (CD45^+^TCRβ^−^CD11b^+^F4/80^+^) isolated by Cell Sorter SH800S from the colonic LP of sham-treated or T cell-transferred mice using the RNeasy Mini Kit (Qiagen, Hilden, Germany), according to the manufacturer’s instructions. RT-qPCR was performed using the RNA-to-CT 1-step kit (Thermo Fisher Scientific) and QuantStudio 7 Flex Real-Time PCR System (Thermo Fisher Scientific). All primers (*TNFα*: Mm00443258_m1, *p40*: Mm01288989_m1, *IL-6*: Mm00446190_m1, inducible nitric oxide synthase (*iNOS*): Mm00440502_m1, and glyceraldehyde 3-phosphate dehydrogenase (*GAPDH*): Mm99999915_g1) were purchased from Thermo Fisher Scientific. Comparative cycle time (CT) was used to normalize the transcript levels to those of GAPDH.

### Co-culture

T cells (CD45^+^TCRβ^+^CD335^−^; 5 × 10^4^) isolated by Cell Sorter SH800S from the colonic LP of T cell-transferred mice were seeded in a 96-well plate in RPMI 1640 supplemented with 10% FBS, penicillin (100 units/mL), and streptomycin (100 units/mL). Then, macrophages (5 × 10^3^) isolated from the colonic LP cells of sham-treated or T cell-transferred mice were added to each well. After two days, the supernatants were collected and stored at − 80 °C until they were assayed. Levels of interferon (IFN)-γ (R&D Systems) and IL-17A (R&D Systems) were determined using ELISA kits, according to the manufacturer’s instructions.

### Statistical analyses

Significance of the average differences between two groups was evaluated using the F-test, followed by the Student’s or Aspin–Welch *t*-test. Significance of the average differences among multiple groups was evaluated using Bartlett’s test, followed by Dunnett’s or Steel’s test. Differences in disease severity were evaluated using Steel’s test. Statistical significance was set at *p* < 0.05.

## Results

### Colonoscopic and histological observations of colitis development

**Fig. 1:**
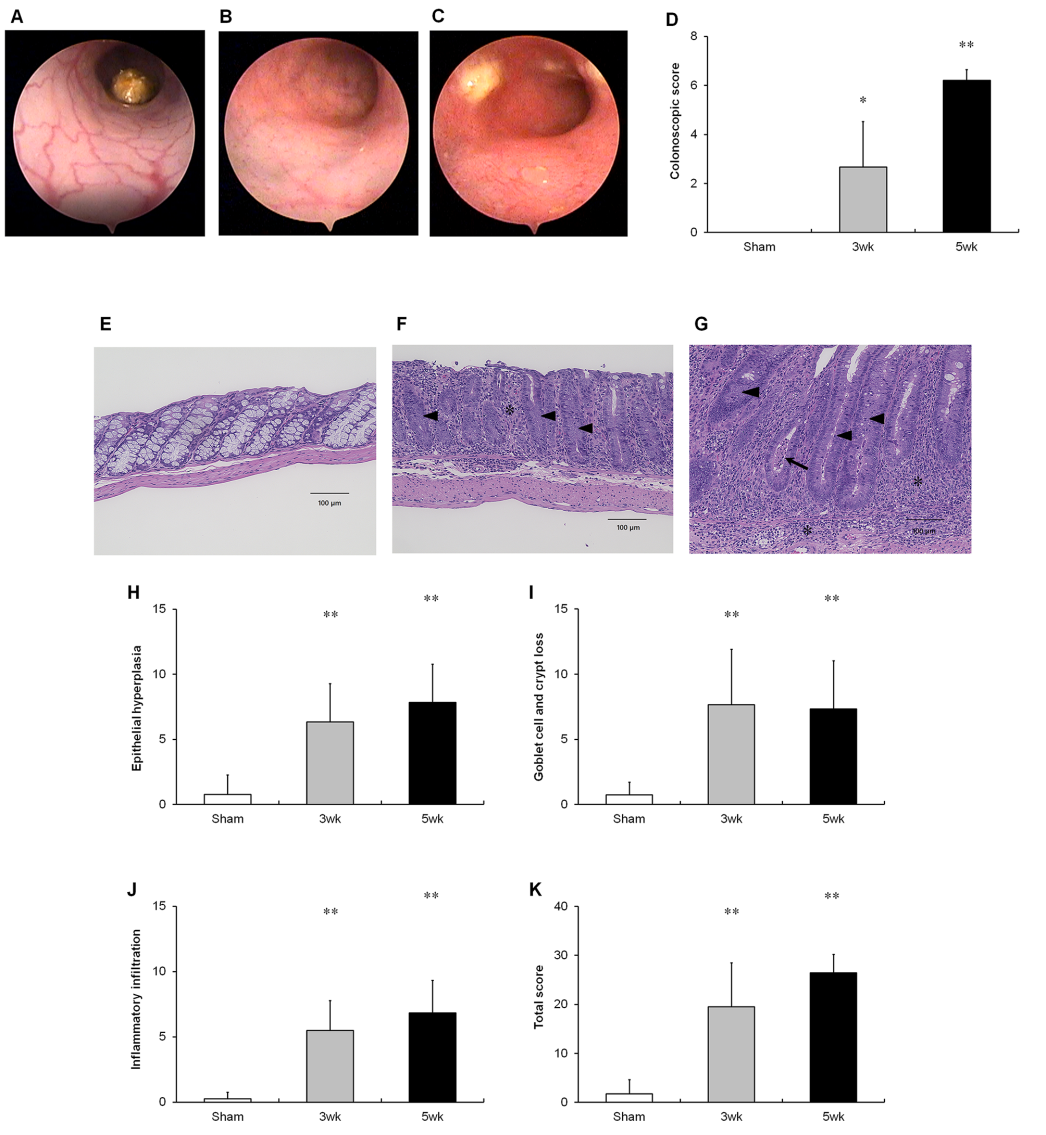
Development of colitis in the T cell transfer colitis model. (**A–C**) Representative colonoscopic pictures of the colon tissues of sham-treated mice (**A**) 3 (**B**) and 5 (**C**) weeks after T cell transfer. (**D**) Colonoscopic scores of sham-treated mice 3 and 5 weeks after T cell transfer. (**E–G**) Representative microphotographs of the colon tissues of sham-treated mice (**E**) 3 (**F**) and 5 (**G**) weeks after T cell transfer. Scale bar, 100 μm. Results are expressed as the mean ± SD. **p < 0.01 and *p < 0.05 via Steel’s test vs. sham-treated group. n = 5–8 animals per group. Immune cell infiltration (asterisk), goblet cell and crypt loss (arrowhead) and crypt abscess (arrow). (**H–K**) Histological scores of sham-treated mice 3 and 5 weeks after T cell transfer. Epithelial hyperplasia (**H**), goblet cell and crypt loss (**I**), inflammatory infiltration (**J**), and total histological score (**K**)

Scid mice started to develop clinical signs of colitis, including weight loss with loose stool and/or diarrhea, three weeks after the adoptive transfer of CD4^+^CD45RB^hi^ T cells. Colonoscopy was performed to monitor the signs of mucosal inflammation. Higher scores observed at 3 and 5 weeks after T cell transfer than in the sham-treated mice (Fig. 1A–D). Moreover, histological changes in the colon were observed three weeks after adoptive transfer. Mucosal and submucosal infiltration of inflammatory cells gradually increased and was associated with a significant increase in epithelial hyperplasia and goblet cell loss as the disease progressed from 3 to 5 weeks (Fig. 1E–K).

### Colonic inflammation

**Fig. 2:**
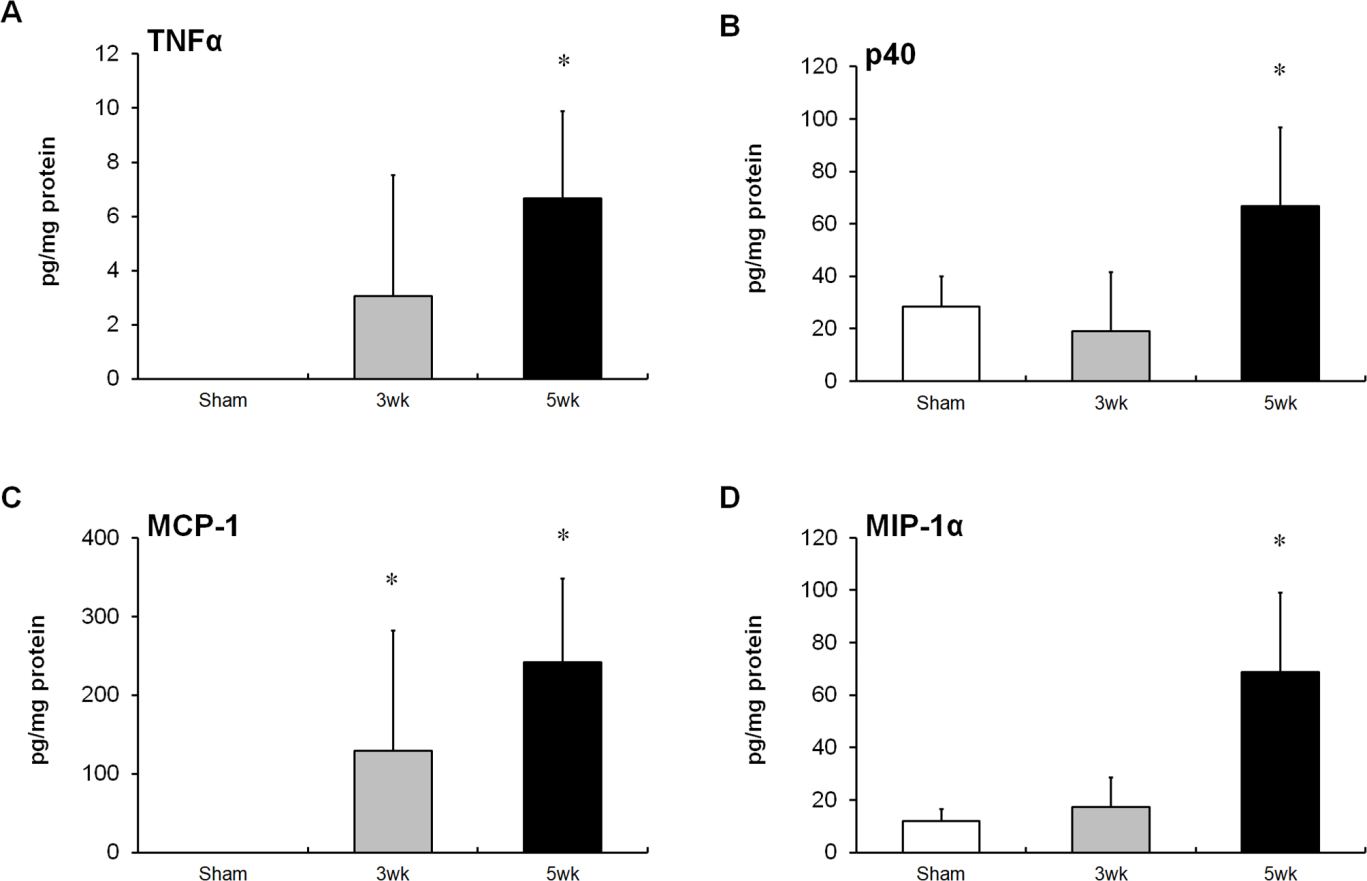
Inflammatory cytokine levels in the colon tissues of the T cell transfer colitis model. ELISA kits were used to measure the tumor necrosis factor (TNF)-α (**A**), p40 (**B**), MCP-1 (**C**), and MIP-1α (**D**) levels in the colon tissues of mice. Results are expressed as the mean ± SD. *p* < 0.01 via Dunnett’s test vs. sham-treated group. n = 5–8 animals per group

**Fig. 3:**
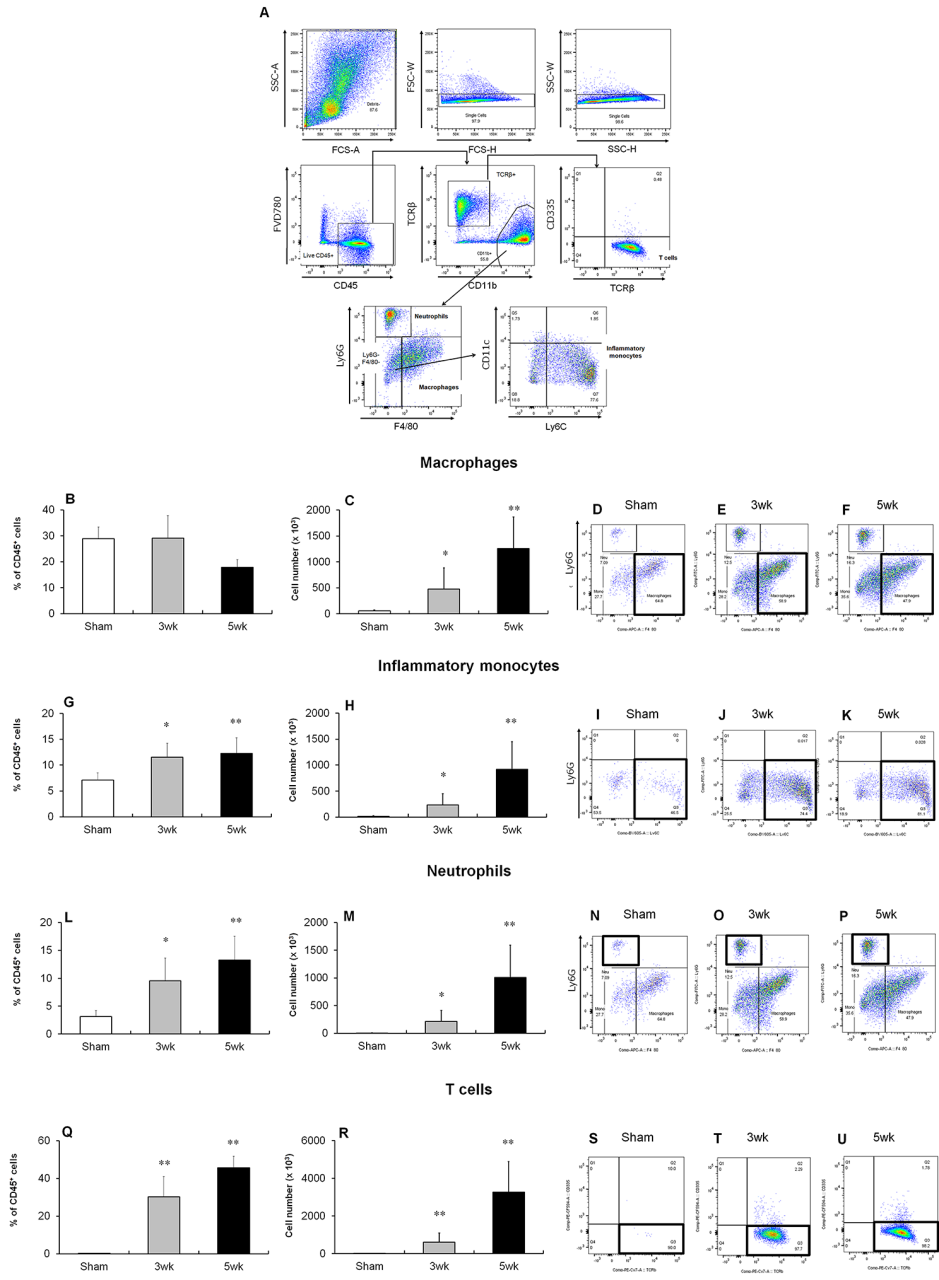
Association of macrophages in the colonic LP of the T cell transfer colitis model. (**A**) Gating strategy for the analysis of LP cells. (**B–U**) Percentage of macrophages (**B**), Inflammatory monocytes (**G**), neutrophils (**L**) and T cells (**Q**) in CD45^+^ cells of colonic LP cells. Numbers of macrophages (**C**), inflammatory monocytes (**H**), neutrophils (**M**), and T cells (**R**) of colonic LP cells. Results are expressed as the mean ± SD. **p < 0.01 and *p < 0.05 via Dunnett’s or Steel’s test vs. sham-treated group. n = 5–8 animals per group. Representative flow cytometric plots of the colonic LP cells of sham-treated mice (**D, I, N and S**) 3 (**E, J, O and T**) and 5 (**F, K, P and U**) weeks after T cell transfer. The area enclosed by the black square indicates macrophages (**D–F**), inflammatory monocytes (**I–K**), neutrophils (**N–P**), and T cells (**S–U**)

**Fig. 4:**
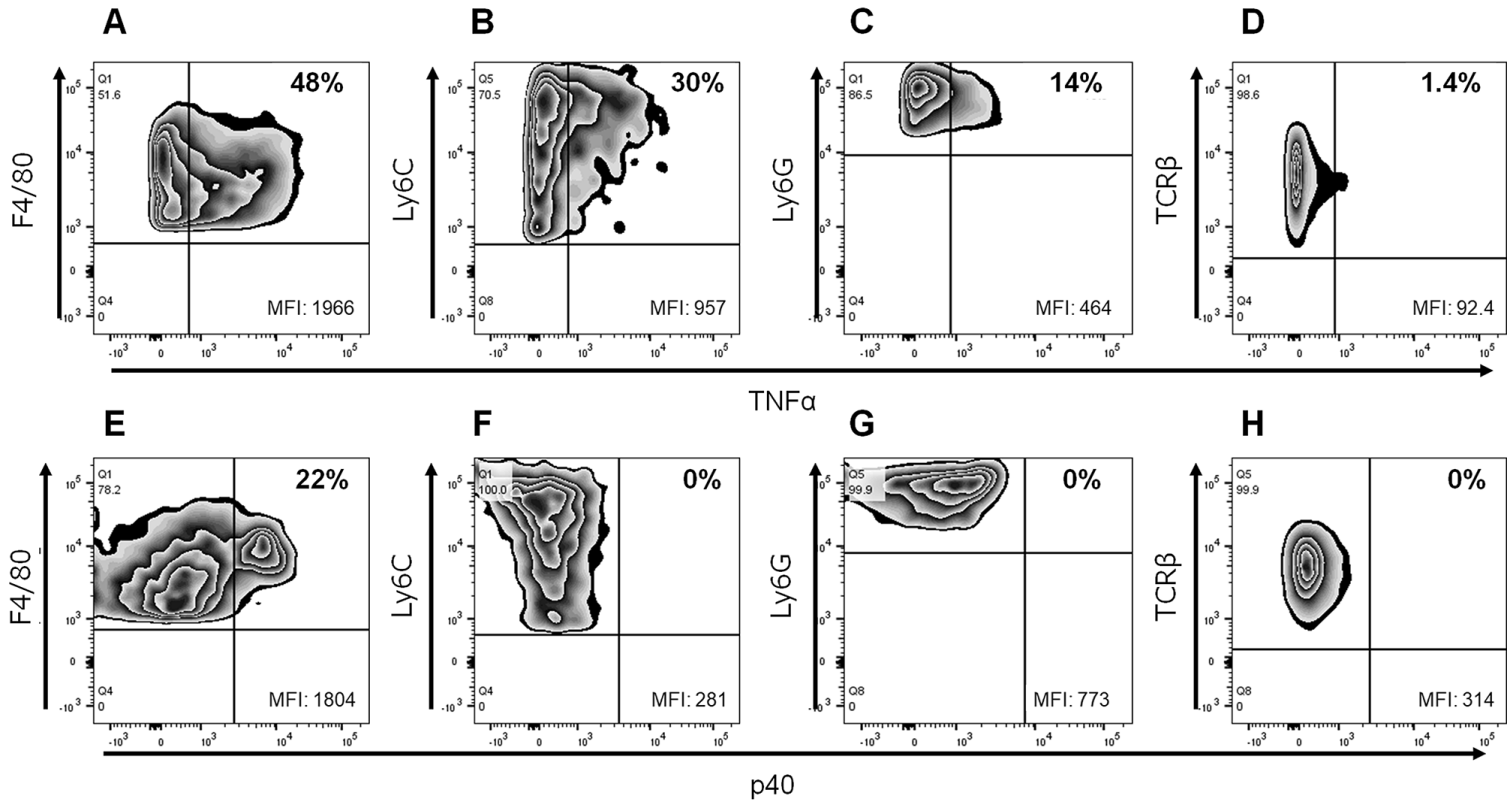
Inflammatory cytokines in macrophages in the colonic LP of the T cell transfer colitis model. (**A–D**) Representative flow cytometric plots of TNFα-producing cells, namely macrophages (**A**), inflammatory monocytes (**B**), neutrophils (**C**), and T cells (**D**), in the colonic LP. (**E–H**) Representative flow cytometric plots of p40-producing cells, namely macrophages (**E**), inflammatory monocytes (**F**), neutrophils (**G**), and T cells (**H**), in the colonic LP. MFI, mean fluorescence intensity

Next, we quantified the colonic protein levels of inflammatory markers in the colitis model. After adoptive transfer, production of pro-inflammatory cytokines, such as TNFα (Fig. 2A) and p40 (Fig. 2B), and macrophage-related chemokines, such as MCP-1 (Fig. 2C) and MIP-1α (Fig. 2D), were increased with colitis development. Flow cytometry was used to evaluate the infiltration of immune cells into the colonic LP of model mice (Fig. 3A). Notably, the numbers of macrophages (Fig. 3B–F), inflammatory monocytes (Fig. 3G–K), neutrophils (Fig. 3L–P), and T cells (Fig. 3Q–U) in the colon increased with colitis development. The percentage of macrophages in the intestine did not change (Fig. 3B), indicating that other infiltrating immune cells, including T cells and neutrophils, increased more than macrophages during the intestinal inflammation. To identify the cellular sources of pro-inflammatory cytokines, such as TNFα and p40, secreted in the colon five weeks after transfer, we performed intracellular TNFα and p40 staining in combination with various cell surface markers using flow cytometry. TNFα was mainly found to originate from macrophages and also from other cells, such as inflammatory monocytes and neutrophils (Fig. 4A–D). However, p40 originated only from macrophages (Fig. 4E–H). Taken together, our results indicate that macrophages in the colonic LP facilitate the development of colitis via the production of inflammatory cytokines, such as TNFα and p40, in the T cell-transfer colitis model.

### Phenotypes and functions of macrophages during intestinal inflammation

**Fig. 5:**
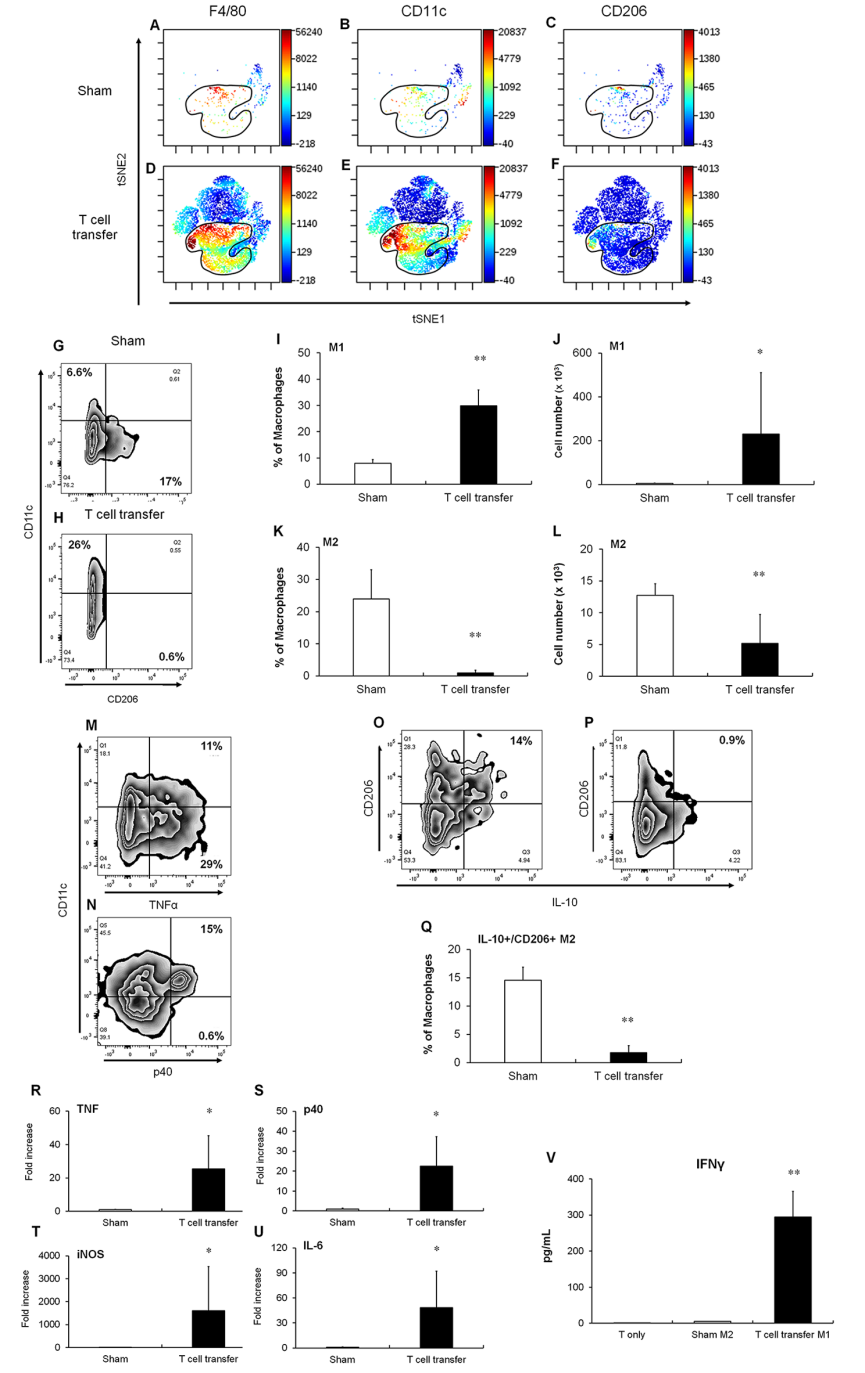
Association of M1/M2 macrophages in the colonic LP of the T cell transfer colitis model. (**A-F**) viSNE plots of the LP cells of sham-treated (**A–C**) and T cell-transferred (**D–F**) mice. F4/80^+^ (**A and D**), CD11c^+^ (**B and E**) and CD206^+^ (**C and F**). Encircled area indicates F4/80^+^ macrophages. (**G and H**) M1 (CD11c^+^) and M2 (CD206^+^) macrophages in the colonic LP of sham-treated (**G**) and T cell-transferred (**H**) mice. (**I–L**) Percentage of M1 (**I**) and M2 (**K**) macrophages in the total macrophages, and the numbers of M1 (**J**) and M2 (**L**) macrophages in the colonic LP of the colitis model. (**M and N**) The expressions of TNFα (**M**) and p40 (**N**) in M1 (CD11c^+^) macrophages in the colonic LP of T cell-transferred mice. (**O and P**) The expressions of IL-10 in M2 (CD206^+^) macrophages in the colonic LP of sham-treated (**O**) and T cell-transferred (**P**) mice. (**Q**) Percentage of IL-10^+^CD206^+^ cells in the total macrophages in the colonic LP of the colitis model. Results are expressed as the mean ± SD. **p < 0.01 via Student’s *t*-test vs. sham-treated group. n = 4 animals per group. (**R–U**) Expression levels of M1 markers in isolated macrophages of the colonic LP of sham-treated and T cell-transferred mice. Transcript levels of TNFα (**R**), p40 (**S**), iNOS (**T**), and IL-6 (**U**). Results are expressed as the mean ± SD. *p < 0.05 via Student’s or Aspin–Welch *t*-test vs. sham-treated group. n = 4 animals per group. (**V**) Effect of the interaction with M1 macrophages on the production of interferon (IFN)-γ protein in the co-culture supernatant. After 48 h, protein levels were determined using ELISA. Results are expressed as the mean ± SD. **p < 0.01 via Dunnett’s test vs. T cell only culture group. n = 3 animals per group

We performed high-dimensional single-cell data analysis by visualizing t-distributed Stochastic Neighbor Embedding (viSNE) of the colonic LP cell populations of sham-treated and T cell-transferred mice to identify the main cell subsets based on the expression levels of F4/80 and M1/M2 markers, such as CD11c and CD206 (Fig. 5A–F). Most CD11c^+^ and all CD206^+^ cells formed separate clusters in the F4/80-positive population in the colons of sham-treated and T cell-transferred mice. Next, we confirmed the changes in the frequencies and numbers of M1 and M2 macrophages with the development of colitis using a gating-based analysis. As shown in Fig. 5G–J, the percentage and number of M1 macrophages (F4/80^+^CD11c^+^) were increased in the colonic LP of T cell-transferred mice than in the sham-treated mice as well as patients with IBD [16, 17]. In mice, CD11c mainly act as the markers of dendritic cells; however, M1 macrophages in the intestine also express CD11c [22–24]. M2 macrophages promote tissue remodeling and maintain gut homeostasis. Macrophages carrying the mannose receptor, CD206, are wound-healing macrophages, which are in the injured mucosa of patients with UC and CD [17, 25, 26]. Here, we observed M2 macrophages (F4/80^+^CD206^+^) in the colons of sham-treated mice (Fig. 5G). The percentage and number of mucosal M2 macrophages (F4/80^+^CD206^+^) in the colonic LP of T cell-treated mice decreased with the development of colitis (Fig. 5G, H, K, and L). Collectively, our results indicate that macrophages in the LP transition from the M2 to M1 phenotype during intestinal inflammation.

Next, we analyzed the ability of M1 macrophages to secrete TNFα and p40, which are detected in colonic tissues during colitis development (Fig. 2A and B). We found that one-fourth of TNFα-producing macrophages and almost all of p40-producing macrophages were M1 macrophages (Fig. 5M and N). M2 macrophages release IL-10, an anti-inflammatory cytokine, in a steady state [27]. Flow cytometric analysis revealed that IL-10-producing macrophages observed in the colonic LP of sham-treated mice were M2 macrophages (Fig. 5O) and that percentage of colonic IL-10-producing M2 macrophages decreased with the development of colitis (Fig. 5O–Q). Furthermore, the expression levels of inflammatory markers, including TNFα, p40, IL-6, and iNOS, were upregulated in the colonic macrophages of T cell-transferred mice than in those of sham-treated mice (Fig. 5R–U). To investigate the ability of colonic macrophages of T cell-transferred mice to activate T cells, macrophages were co-cultured with T cells isolated from the colonic LP of T cell-transferred mice for 48 h. Activated T cells produce IFNγ and/or IL-17 A in the colonic LP of the T cell transfer colitis model [11, 12]. Here, IFNγ was produced by T cells co-cultured with macrophages in the LP of T cell-transferred mice, but not in sham-treated mice (Fig. 5V). In contrast, IL-17 A was not produced by T cells (Additional file 1). Taken together, M1 macrophages in the colonic LP play critical roles in the production of inflammatory cytokines and activation of colitogenic T cells during colitis development.

## Discussion

Macrophages regulate intestinal immune homeostasis by distinguishing harmless antigens from potential pathogens and maintaining oral tolerance. Recently, specific molecules and cellular mechanisms involved in the rescue of inflammation have been identified. Particularly, macrophages play key roles in the prevention of exaggerated immune responses [28]. In the early phase of IBD, the infiltrating M1 macrophages engulf foreign pathogens and clear bacteria and cellular debris from wound. However, the secretion of more inflammatory cytokine, including TNFα and p40, and high levels of iNOS and reactive oxygen species ultimately aggravate the inflammatory response and also cause tissue damage and impaired wound healing [15–17]. Therefore, therapeutic targeting of macrophages may be an effective strategy to maintain the intestinal immune microenvironment and restore tissue homeostasis after inflammation [15]. However, their specific contributions to the pathogenesis of IBD remain unclear, increasing the difficulty of developing macrophage-targeted therapies.

In this study, we assessed the functions of intestinal macrophages in a T-cell transfer colitis model. M1 macrophages are the main cellular sources of inflammatory cytokines, such as TNFα and p40, which are indispensable for the development of colitis. We found that the number of intestinal M1 macrophages increased, whereas that of M2 macrophages decreased during colitis development in mice as observed in patients with IBD [16]. It is considered that mucosal infiltrating inflammatory monocytes differentiate to M1 macrophages and M2 macrophages transition to M1 macrophages during the mucosal inflammation development in mice. As the results, the number of mucosal M1 macrophages increase, whereas that of mucosal M2 macrophages decrease during colitis development. Classically activated M1 macrophages have a pro-inflammatory phenotype, whereas alternatively activated M2 macrophages have an anti-inflammatory phenotype and promote tissue repair [29, 30]. However, the surface markers identified in vitro-generated macrophages do not translate to the macrophages in vivo [31]. Here, macrophages in the colonic LP of sham-treated and T cell-transferred mice expressed the M1/M2 markers, CD11c and CD206, respectively, which are also observed in patients with IBD [16, 17, 27]. IL-10 is an anti-inflammatory cytokine that plays a crucial role in gut homeostasis. CD206^+^ M2 macrophages in the human colon express higher levels of IL-10 transcripts than CD206^−^ macrophages [26]. In this study, CD206^+^ M2 macrophages in the colon of sham-treated mice produced IL-10, and their number decreased with the onset of colitis. Therefore, IL-10-producing M2 macrophages in steady state are crucial for the regulation of mucosal homeostasis in mice as well as in human [26].

T cell activation is essential for the development of colitis in T cell transfer models [11–13]. Interestingly, in our co-culture system, T cells isolated from the colonic LP produced IFNγ, but not IL-17 A, by interacting with M1 macrophages. IFNγ and/or IL-17 A production by T cells is necessary for colitis development [11, 12]. T cell activation in the colonic LP occurs in response to intestinal microbiota, such as CBir1 flagellin-expressing *Clostridia* [35] or *Helicobacter* species [36], in T cell transfer colitis models. Here, mucosal IFNγ-producing T cell (Th1 or Th1/17 cells) activation required interaction with the infiltrated M1 macrophages in colon under intestinal inflammation. However, the mechanism of T-cell activation by M1 macrophages remains unclear and requires further investigation. Nevertheless, our findings suggest that T cell activation and interaction with M1 macrophages play indispensable roles in the pathophysiology of human IBD. Recently, targeting macrophage polarization has shown great potential for the treatment of IBD [32–34]. For instance, resolvin and other mediators known to be involved in resolution of inflammation can direct macrophage differentiation towards an M2 phenotypes [32, 34]. As another example, mannose-modified trimethyl chitosan-conjugated nanoparticles can inhibit the activation of M1 macrophages and induce the phenotype of M2 macrophages by suppressing the TLR4 signaling pathway [32]. Although some differences are observed in the functions of macrophages between mice and humans [37], this study provides useful information for the elucidation of the in vivo functions of macrophages in the colonic LP of patients with IBD.

## Conclusions

In this study, we found that the expression patterns of M1 and M2 markers in a T cell transfer colitis model could be identified as in the patients with IBD and that the macrophages polarized toward the M1 phenotype in the colonic LP of the colitis model. Furthermore, M1 macrophages activated the colitogenic T cells. Our findings suggest the T cell transfer model as a useful tool for the elucidation of mucosal macrophage functions in human IBD, and for the efficacy evaluation of not only T cell-targeted but also macrophage-targeted (especially macrophage polarization-targeted) drugs.

### Electronic supplementary material

Below is the link to the electronic supplementary material.

Supplementary Material 1: IL-17A was not produced by T cells co-cultured with M1 macrophages in the LP of T cell-transferred mice

## Data Availability

All data that support the findings of this study are available upon request from the corresponding author.

## Abbreviations

CD: Crohn’s disease
CT: Comparative cycle time
DAMPs: Damage-associated molecular patterns
ELISA: Enzyme-linked immunosorbent assayl
FBS: Fetal bovine serum
FMO: Fluorescence minus one
FVD780: Fixable Viability Dye eFluor 780
IBD: Inflammatory bowel disease
IFN: Interferon
IL: Interleukin
iNOS: Inducible nitric oxide synthase
LP: Lamina propria
MCP-1: Monocyte chemoattractant protein-1
MFI: Mean fluorescence intensity
MIP: Macrophage inflammatory protein
PAMPs: Pathogen-associated molecular patterns
scid: C.B-17/Icr-Prkdc < scid>/CrlCrlj
TNF: Tumor necrosis factor
UC: Ulcerative colitis
viSNE: t-Distributed Stochastic Neighbor Embedding
