# Supplementary material for: Macrophage polarization toward M1 phenotype in T cell transfer colitis model
Source: BMC Gastroenterol. 2023 Nov 27;23:411. doi: 10.1186/s12876-023-03054-1 (PMC10680295; doi:10.1186/s12876-023-03054-1)
Supplement: Supplementary file 1 — Supplementary Material 1: IL-17A was not produced by T cells co-cultured with M1 macrophages in the LP of T cell-transferred mice [file 12876_2023_3054_MOESM1_ESM.pdf]

Effect of the interaction with M1 macrophages on the production of IL-17A in the coculture supernatant.
After 48 h, protein levels were determined using ELISA.

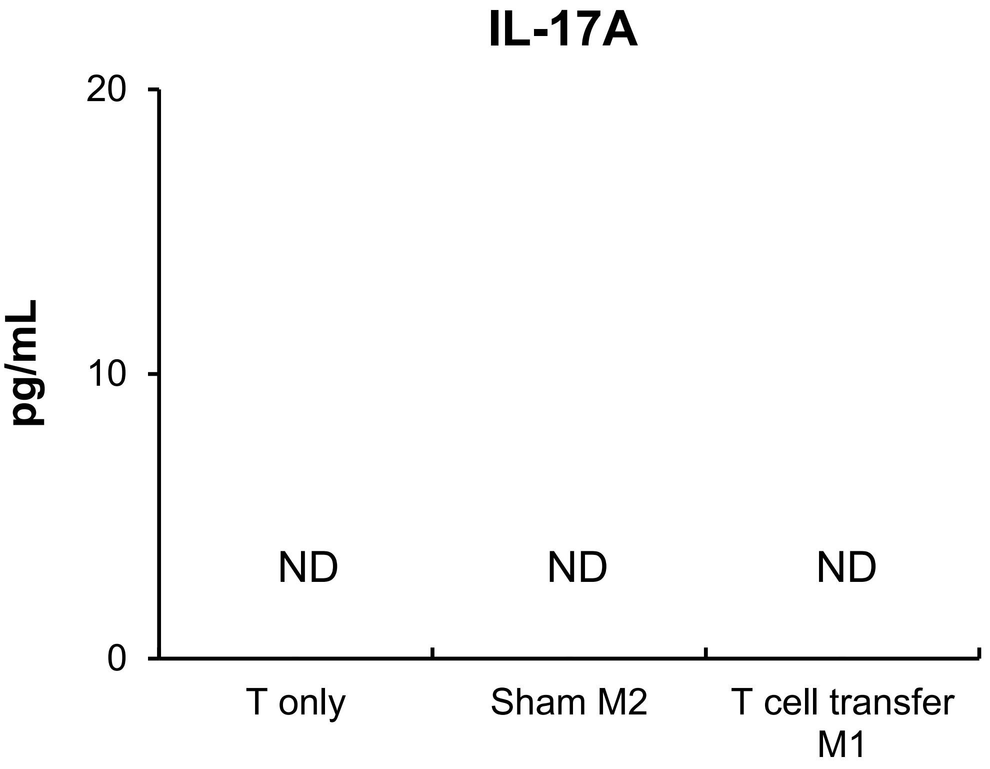

Additional file 1
